# Supplementary figures and images for: Robust Transcriptional Profiling and Identification of Differentially Expressed Genes With Low Input RNA Sequencing of Adult Hippocampal Neural Stem and Progenitor Populations
Source: Front Mol Neurosci. 2022 Jan 31;15:810722. doi: 10.3389/fnmol.2022.810722 (PMC8842474; doi:10.3389/fnmol.2022.810722)

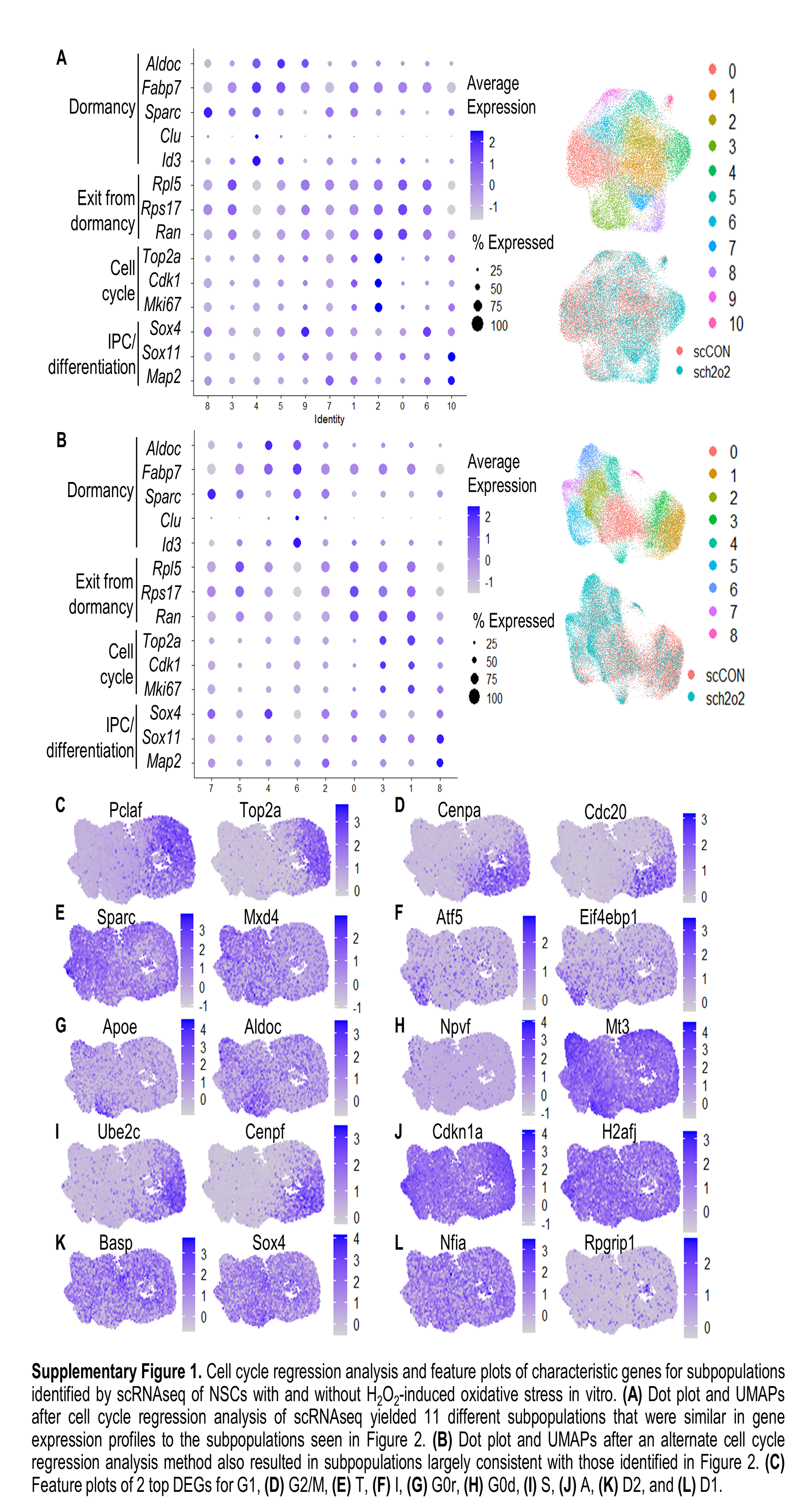

Supplement: Supplementary file 1 [file Image_1.TIF]

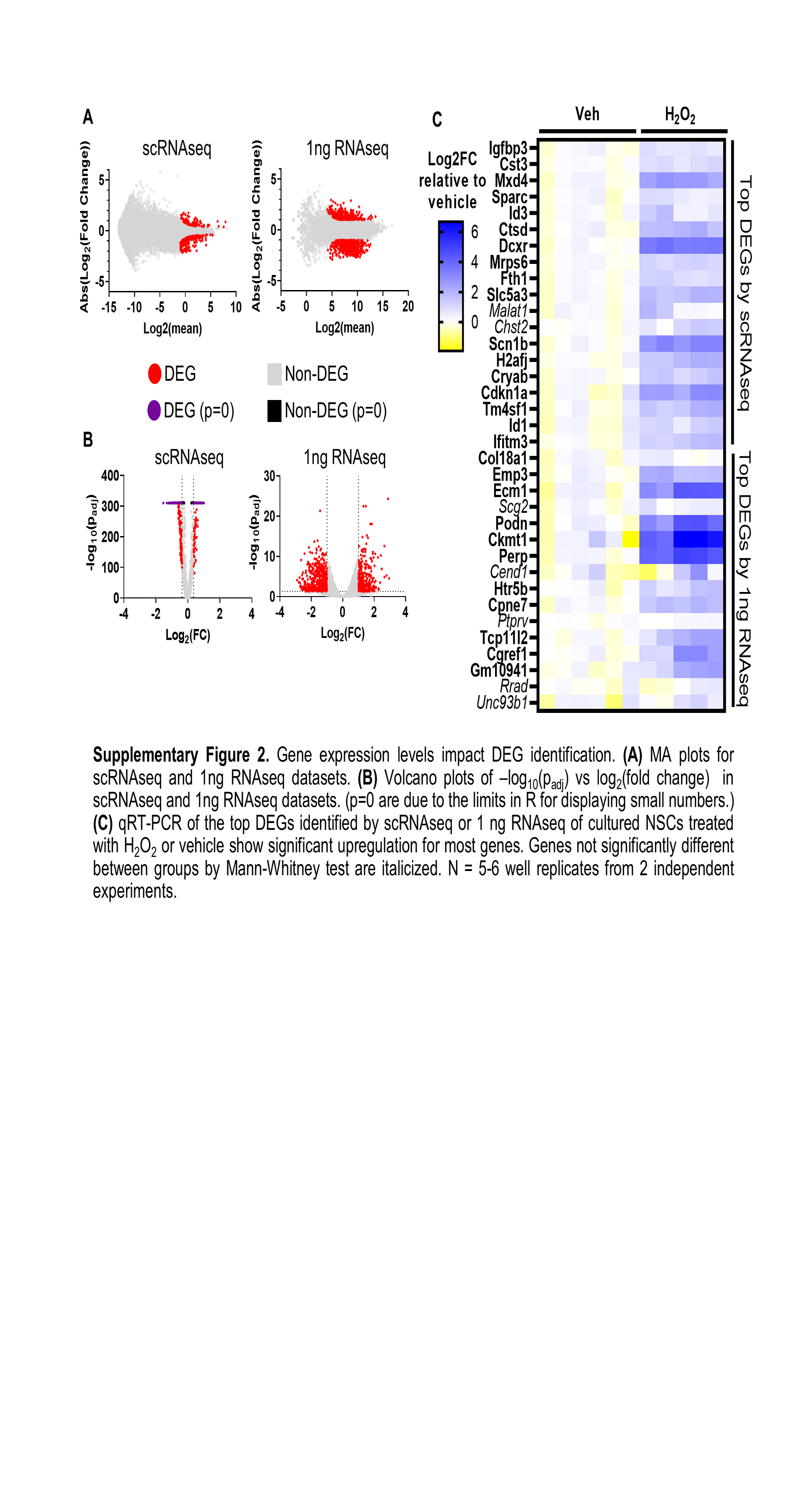

Supplement: Supplementary file 2 [file Image_2.TIF]

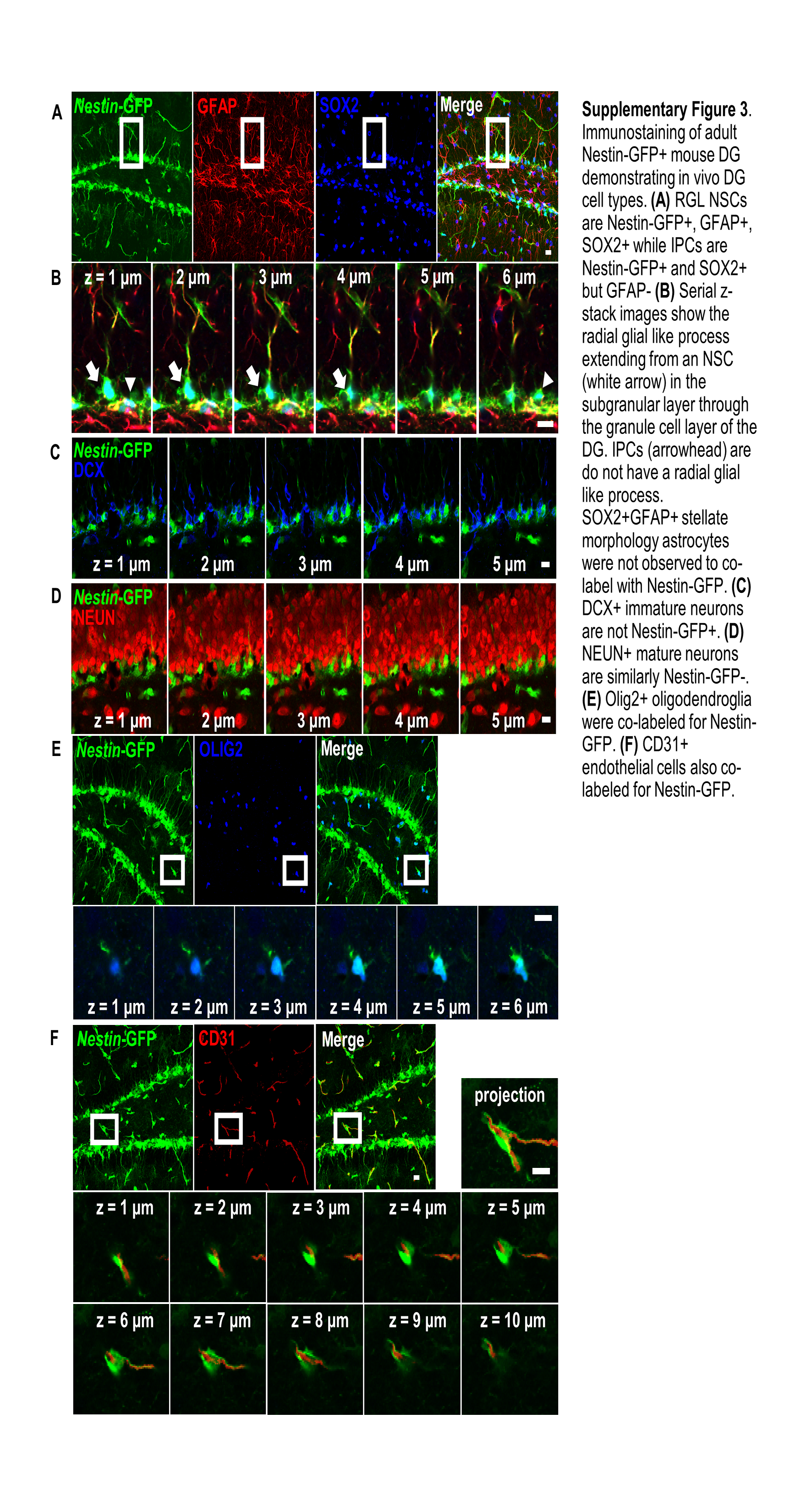

Supplement: Supplementary file 3 [file Image_3.TIF]

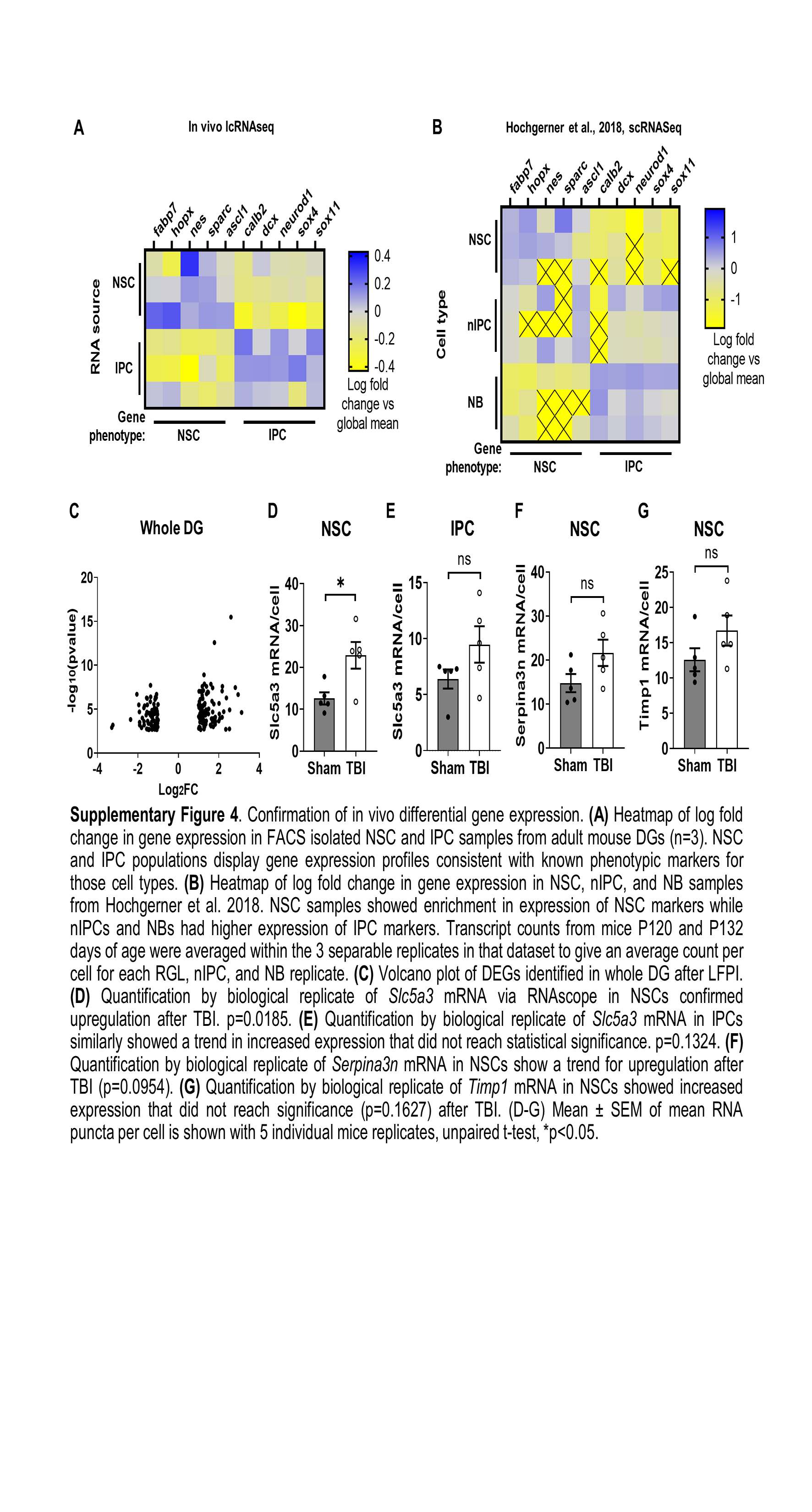

Supplement: Supplementary file 4 [file Image_4.TIF]
